# Unmet Medical Needs of Patients with Benign Prostate Enlargement

**DOI:** 10.3390/jcm9040895

**Published:** 2020-03-25

**Authors:** Munjae Lee, Sewon Park, Mankyu Choi, Kyu-Sung Lee

**Affiliations:** 1 Department of Medical Device Management and Research, SAIHST, Sungkyunkwan University, Seoul 06351, Korea; emunjae@skku.edu (M.L.); se10919@g.skku.edu (S.P.); 2 Department of Public Health Science, Graduate School of Korea University, Seoul 02841, Korea; 3 BK21Plus Program in Public Health Science, Korea University, Seoul 02841, Korea; 4 Department of Urology, Samsung Medical Center, Sungkyunkwan University School of Medicine, Seoul 06351, Korea

**Keywords:** benign prostate enlargement, benign prostatic hyperplasia, unmet medical needs, Andersen’s behavioral model, community care, Korea

## Abstract

This study aimed to analyze the factors affecting the unmet medical needs of patients with benign prostate enlargement (BPE) based on Andersen’s behavioral model. The data were taken from the 2009–2016 Korea Health Panel Study and 3003 participants were used for analysis. “Unmet medical needs” was used as a dependent variable. Independent variables were predisposing variables: age, educational attainment, and marital status; enabling factors: income, job type, and insurance type; and need factors: lying in a sickbed, activity limitation, subjective health status, and having chronic diseases. Results showed that younger patients experienced a higher probability of unmet medical needs. Those with higher educational attainment had a lower chance of experiencing unmet medical needs. Patients with national health insurance were less likely to experience unmet medical needs. In addition, patients who experienced lying in a sickbed had a higher probability of experiencing unmet medical needs. Therefore, in order to reduce the unmet medical needs of patients with BPE, it is necessary to allow patients to be treated early and give them accurate information about the disease. In addition, access to medical care should be strengthened through continuous care focused on primary care.

## 1. Introduction

Benign prostate enlargement (BPE) is known as a senile disease; its prevalence is rapidly increasing in those in their 40s due to a Westernized dietary life and among patients with metabolic syndromes, such as obesity, diabetes, and hypertension [1,2]. BPE, one of the most common diseases in middle-aged men, is a representative disease that causes lower urinary tract symptoms and the enlargement of the prostate gland, which blocks the urethra and induces urinary disorders. BPE, which occurs due to aging and an imbalance of male hormones, may lead to serious complications, such as urethrostenosis, bladder stones, hematuria, acute urinary retention, renal failure, etc., which lowers patients’ quality of life [3,4,5].

BPE is not a life-threatening disease, but one that reduces patients’ quality of life due to the lower urinary tract symptoms, which have negative physical and psychological effects. This leads to the shortening of patients’ health span and a decrease in their economic activity, resulting in individual social and economic losses. BPE is an illness that cannot be completely cured, and it requires continuous care [6,7,8]. The increased prevalence of BPE causes an increase in medical expenses; it has become difficult to balance continuously rising medical expenses and meeting medical needs [9,10].

The inability to satisfy a patient’s medical needs is referred to as unmet health care. Unmet medical care means that although there is a medical need, the necessary medical services are not received or available in time, due to various factors, such as social and economic conditions. If the patient was not treated in a timely manner, owing to the occurrence of unmet medical care, it might increase the possibility of the patient acquiring moderate degree diseases and their accompanying complications. Since preventing unmet medical care helps to alleviate symptoms, as well as to prevent and treat diseases, it is considered a medical problem that needs to be solved [11,12]. Andersen’s behavioral model is used to analyze the predictors of medical service use and how these affect patient outcomes, such as health condition and health satisfaction. Furthermore, it defines a fair approach to health care and measures factors that affect medical use, so it may also be applied to research on unmet medical needs.

Since BPE is a disease that needs to be treated and managed, proper information about the disease needs to be provided so that an appropriate diagnosis and treatments may be received. In addition, because the symptoms of BPE are thought to be caused by aging, and thus not treated in a timely manner, there are difficulties in the treatment of the disease [13,14]. Therefore, this study intends to investigate the current condition of unmet medical care in patients with BPE and to analyze the factors affecting their unmet medical care based on Andersen’s behavioral model. Through these, we are going to provide the baseline data to improve the quality of medical services for patients with BPE.

## 2. Materials and Methods

### 2.1. Subjects and Data Sources

We used data from the Korea Health Panel Study (KHPS). The KHPS has been jointly surveyed by the Korea Institute for Health and Social Affairs and the National Health Insurance Service yearly since 2008; it has been utilized as the baseline data for the establishment and enforcement of health care policies in preparation for aging. We intended to analyze the factors affecting unmet medical care using the data from the KHPS over eight years, from 2009 to 2016. Therefore, 155,521 people who participated in the KHPS were selected as the research subjects. Since BPE is a common disease in middle-aged males, 16,443 male patients who had been diagnosed with BPE and received medical care services were included as the research subjects. The patients with BPE, the subjects were classified only by the presence or absence of BPE diagnosis. The subjects (*n* = 827) who did not respond to the questions measuring whether they had ever experienced unmet medical care were excluded. Finally, in terms of Andersen’s behavioral model, 3003 people were utilized in the analysis after excluding the subjects (*n* = 2293) who did not respond to the questions related to the predisposing factors, those (*n* = 4344) who did not respond to the questions associated with the enabling factors, and those (*n* = 5976) who did not respond to the questions related to the need factors. The KHPS is approved by the ethical committee of the Korea Institute for Health and Social Affairs and the National Health Insurance Service. The requirement for informed consent was waived because data in the KHPS are anonymized in adherence to strict confidentiality guidelines.

### 2.2. Variable Definition

#### 2.2.1. Dependent Variable

The dependent variable in this study is the occurrence of unmet medical care. The measurement of unmet medical care was performed using the question, “Did you experience not being able to receive medical treatments or tests at a clinic or hospital over the past year?”, from a survey conducted by the KHPS. Only respondents that answered that they had experienced not receiving medical care services at least once were encoded as “1” and those that responded they had never experienced unmet medical care were classified as “0”.

#### 2.2.2. Independent Variable

The independent variables of this study were constructed based on Andersen’s behavioral model; it categorizes the factors related to why medical care services are sought by dividing them into predisposing factors, enabling factors, and need factors in order to predict the factors affecting medical use. The predisposing factors refer to the basic factors an individual has, such as gender, age, marital status, and education level. The enabling factors are related to the resources of patients, including material factors, such as household income, insurance type, social capital, etc. Disease factors refer to variables representing medical needs, including the patients’ subjective health status, number of activity-limited days, the experience of lying in a sickbed, and whether they have chronic diseases and complications [15,16,17].

In this study, the predisposing factors included age, education level, and marital status. Age was analyzed by dividing respondents into groups aged above or below 65 years old. Education level was classified on the basis of high school graduation. With regard to marital status, those currently married and in a de facto marriage were categorized into “having a spouse” while those separated, bereaved, and never married were classified into “no spouse.” Enabling factors included household income, job type, and insurance type. Household income was classified based on whether gross annual household income exceeded three million won. Job types were divided into paid work and self-employment. The insurance type was classified into health insurance subscribers and medical beneficiaries. Need factors consisted of experiencing lying in a sickbed, activity limitation, subjective health status, and having a chronic disease. Patients who have had to lie down almost all day due to an illness or injury over the last month were classified as having the experience of lying in a sickbed. With regard to activity limitation, the cases that responded “Yes” to the item, “I have experienced activity limitations due to illness, injury, or the like”, were classified as having activity limitations. The questions measuring subjective health status were used in the analysis. Patients who have at least one chronic disease were classified as chronic disease patients. For the items with five-point scales, “Very good” and “Good” were classified as “Good”, and “Normal” to “Very bad” were classified as “Bad.”

### 2.3. Research Model

**Figure 1:**
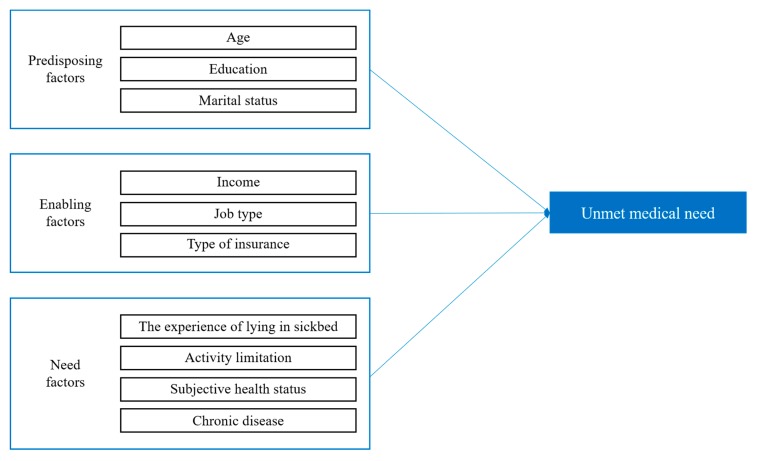
Research model.

The data collected in this study were analyzed using the SPSS 25.0 program. First, frequency analysis was performed to analyze the unmet status for patients with BPE and to identify their sociodemographic characteristics. Second, cross-tabulation analysis was conducted to analyze the differences in the occurrence of unmet medical care according to the subjects’ predisposing factors, enabling factors, and need factors. Third, hierarchical logistic regression analysis was performed to analyze the factors influencing the unmet medical care status of patients with BPE. The model of this study is shown in Figure 1.

## 3. Results

The 3003 patients with BPE were the subjects of this study; their general characteristics are shown in Table 1. The average age of patients under 65 was 50.35 years, and the average age of patients over 65 was 77.49 years. In terms of age, 2154 patients (71.7%) were younger than 65 years old and 849 patients (28.3%) were at least 65 years old; a higher percentage of patients were younger than 65 years old. Based on educational attainment, 622 people (20.7%) were not high school graduates and 2381 people (79.3%) graduated at least high school. In terms of marital status, 2556 patients (85.1%) had a spouse and 447 patients (14.9%) did not have a spouse, showing that most patients lived with their spouses. With regard to income, 1885 patients (61.8%) monthly earned less than three million won, and 1118 patients (37.2%) earned more than three million won. In terms of job type, 1855 people (61.8%) worked for wages, and 1148 people (38.2%) were self-employed workers, suggesting that most patients worked for wages. As for the insurance type, 2961 people (98.6%) had health insurance and 42 people (1.4%) were medical beneficiaries. Two hundred fifty-seven patients (8.6%) had experienced lying in a sickbed, whereas 2746 people (91.4%) had not. In addition, 105 patients (305%) experienced activity limitations due to a disease, but 2898 patients (96.5%) did not. Regarding subjective health status, 2415 patients (80.4%) considered their health to be good, whereas 588 patients (19.6%) considered it to be bad. Meanwhile, 989 patients (32.9%) with BPE also had chronic diseases and 2014 patients (67.4%) did not have chronic diseases. Eight hundred twenty-four patients (27.4%) experienced unmet medical care and 2179 (72.6%) did not.

Cross-tabulation analysis was performed to analyze the relationship between the unmet medical needs incidence and factors influencing the medical service use of patients with BPE. The result is shown in Table 2. First, in the Anderson model, cross-tabulation analysis between predisposing factors, which include age, education, and marital status, and whether unmet medical care may occur, showed education level as the statistically significant factor. Of the patients who did not complete a high school education, 199 patients (32.0%) experienced unmet medical care; while 625 patients (26.2%) whose education level was at least a high school graduate also experienced unmet medical care.

Second, cross-tabulation analysis between enabling factors, which included income, job type, and insurance type, and whether unmet medical care may occur, showed all factors were statistically significant. Among the low-income patients, 542 patients (28.8%) experienced unmet medical care as well as 282 (25.2%) high-income patients, suggesting that low-income patients experience more unmet medical care. In addition, based on job type, 489 people (26.4%) experienced unmet medical care among the patients working for wages, and 335 people (29.2%) among the self-employed patients, which shows that the patients working for wages experience more unmet medical care. Of the patients with BPE who had health insurance, 805 people (27.5%) experienced unmet medical care, while 19 patients (45.2%) who are medical beneficiaries experienced unmet medical care.

Lastly, cross-tabulation analysis between the need factors, such as experiencing lying in a sickbed, activity limitation, subjective health status, and chronic disease, and whether unmet medical care may occur, showed the experience of lying in a sickbed was statistically significant. Of the patients who experienced lying in a sickbed, 54 people (21.0%) experienced unmet medical care; among the patients who have not experienced lying in a sickbed, 770 people (28.0%) experienced unmet medical care, suggesting that patients who have not experienced lying in sickbed experience more unmet medical care. In summary, cross-tabulation analysis showed that the factors related to the unmet medical needs of patients with BPE are education level, income, job type, insurance type, and experience of lying in a sickbed.

Hierarchical logistic regression analysis was performed to investigate the effects of predisposing factors, enabling factors, and need factors on unmet medical care in patients with BPE. The result is shown in Table 3. The analysis sequence was as follows: First, predisposing factors of the patients with BPE, which affect unmet medical care as the dependent variable, were introduced into Step 1 (Model 1). Second, enabling factors were inputted into Step 2 (Model 2), and finally, need factors were introduced into Step 3 (Model 3) to analyze the effect on unmet medical care.

As a result of the analysis, the variables that showed a significant correlation to the predisposing factors (Model 1) were age and education level. As age increased, the probability of experiencing unmet medical care decreased 0.666 times; the higher the educational attainment, the chance of experiencing unmet medical care decreased 0.593 times. Second, in Model 2, where both predisposing factors and enabling factors were inputted together, age and education level, which showed significant results in Model 1, continuously worked as significant variables. Furthermore, of the enabling factors, the insurance type appeared as a significant variable. In other words, for health insurance subscribers, the probability of experiencing unmet medical care decreased 0.476 times. Lastly, in Model 3, which included predisposing factors, enabling factors, and need factors, age, education level, and insurance type continuously emerged as significant variables; while the experience of lying in a sickbed appeared as a need factor affecting unmet medical care. For the patients who had not experienced lying in a sickbed, the chance to experience unmet medical care decreased 0.681 times.

## 4. Discussion

BPE, with a mortality rate of less than 1%, is not a life-threatening disease. However, it induces discomfort by causing urination disorders and other complications; thus, it is a factor in deteriorating quality of life. However, patients do not expect this illness to be improved with treatments as they consider the deterioration of urinary symptoms a product of aging or they do not seek medical attention as they consider it a temporary symptom. According to Andersen’s behavioral model, the factors that have the most direct impact on the patients’ use of health care services are the need factors. That is, the patient first recognizes his or her health status and comes to decide whether to receive medical services based on the degree of seriousness. In the case of BPE, there are likely to be cases where the patient does not receive medical treatment because he does not have a desire to use the medical service, or despite the patient recognizing the disease and feeling a desire to use the medical service, he cannot use the service. Therefore, in this study, we intended to suggest effective management methods for patients with BPE through analysis on what factors may affect the unmet medical treatment of patients with BPE. As a result, in terms of factors affecting unmet medical care in patients with BPE, the predisposing included the factors age and education level, while the enabling factors included insurance type. In addition, from the necessity factors, the experience of lying in a sickbed influenced the unmet medical care of the patients with BPE.

In this study, older patients with BPE had a lower probability of experiencing unmet medical care; this is different from existing studies [18,19,20]. BPE mainly occurs during senescence; the demand for medical care services is increasing due to the increasing elderly population caused by population aging. BPE requires continuous medical care services and careful management. This suggests that the patients with BPE over 65 years of age come to actively receive medical care services, thus reducing the occurrence of unmet medical care. Furthermore, in recent years, there has been an increase in the patients with BPE in their 30s–40s, who simply consider the symptoms of the disease as a discomfort in daily life and thus, seem to have a relatively low desire to use their medical care. In addition, for the elderly, although there are many cases requiring medical treatments due to their high healthcare needs, many patients under 65 years of age often tend to seek treatments for BPE through social networks [21,22].

Unmet medical care is divided into subjects that judge medical needs and fulfillment, and the scope of medical needs. While there are people with medical needs who desire medical care, there are other cases wherein people reject medical care despite having a need for it. In the former case, unsatisfactory medical care occurs because medical use is hindered by the economic burden and medical delivery system. In the latter case, lack of patient awareness and negative attitudes reduce the likelihood of medical use, resulting in unmet medical care. In BPE, subjective judgments make a difference in medical care. Patients under 65 years of age are not aware of the seriousness of medical use and thus have low medical use, and their desire to use medical service is also significantly lower. In particular, many young patients feel naturally healed, which lowers the possibility of medical use, resulting in unmet medical care. It is believed that the wrong treatment information provided via social networks may cause unmet medical care of young patients [23,24]. Therefore, medical providers should strive to develop clinical guidelines that patients can practice and also ensure that patients are fully aware of the content of treatment. Because patients are often reluctant to get BPE treated, it is likely that patient-centered clinical guidelines will increase awareness on the fact that early detection and treatment of the disease can improve it, making medical treatment more acceptable to them. Such health policies and clinical efforts are expected to change social awareness by spreading the correct prevention and treatment information of BPE.

Meanwhile, it was found those with a higher the education level had a lower probability of experiencing unmet medical care. This was consistent with the existing study that with a higher education level comes a better understanding of medical information. Thus, appropriate medical services are provided, and t since the patients with a high education level have higher accessibility to medical care services in solving health problems than those with a low education level, their unmet medical care decreases [25,26,27]. A better understanding of the disease allows for a more accurate provision of treatment, which may reduce the gap between the patient’s medical needs and the health care services they receive; again, decreasing the probability to experience unmet medical care. In particular, as the patients with BPE receive medical care services based on an accurate understanding of symptoms, their medical needs are more satisfied. In addition, in order to meet the medical need of patients with a low education level, it is necessary that the primary medical institutions provide information on the symptoms of BPE to reduce the incidence of unmet medical care.

At present, patients with health insurance do not experience unmet medical care. This is consistent with previous findings that households with health insurance are less likely to have unmet medical care than those receiving medical benefits [26,28,29]. South Korea intends to achieve universal medical security with health insurance and wants to secure medical accessibility for low-income earners through the medical benefit system [30]. However, as households receiving medical benefits have limitations in managing their health care voluntarily due to lack of ability to pay their sharing and other reasons, they are in a situation where they should perform ex post treatment-oriented health care. Households receiving medical benefits may use medical care services after their medical needs occur or receive insufficient medical services due to their problems, such as the burden of medical expenses; thus, resulting in the occurrence of unmet medical care. In terms of BPE, as the symptoms are not life-threatening and the patients do not feel the need to receive medical services even if they are inconvenienced, the occurrence of unmet medical care seems to increase. Therefore, it is necessary to expand the provision of medical services to households receiving medical benefits by reducing the patient’s share for the examination of BPE, reducing non-payment items, etc.

Need factors are the factors that generate more direct needs for medical use, in comparison to predisposing factors and enabling factors. In terms of need factors, it was found that the patients who had not experienced lying in a sickbed did not experience unmet medical care. This was similar to previous studies in which those who had to spend their days lying down due to physical damage caused by diseases or the like had a lower quality of life and lower medical accessibility than those who had not experienced lying in a sickbed [31,32]. The use of medical care is dependent on the subjective health status of the individuals; if patients want to receive medical services, but their medical needs are not satisfied due to their physical activity limitation, unmet medical care will occur. Since the enlarged prostate gland continuously causes symptoms in the lower urinary tract, patients with BPE will receive continuous medication. However, the more time the patients spend in a sickbed, the more likely they will have difficulty receiving continuous medication, which will increase their experiences of unmet medical care. Accordingly, it seems necessary to include the management for patients with BPE in the community care project so that patients can receive care services where they live. If, through the community care project, disease management is available for patients with BPE who have difficulties in visiting medical institutions due to physical limitations, the causes of unmet medical care will be eliminated. In other words, the patients with BPE who are unable to meet their medical needs due to their limited physical activity will also receive medical care at home to resolve unmet medical care. The provision of Information and Communications Technology (ICT)-based medical care and the introduction of care robots for patients with low medical accessibility will also reduce unmet medical care of the patients with BPE. Further, if innovative ICT-based medical devices for community care projects are developed from the results of this study, which identified the demands of patients with unmet medical care suffering from BPE, it may improve the therapeutic effect of treatment. It is thought that this will solve the problem of unmet medical care by providing appropriate medical services to patients with BPE.

This study has the following limitations. First, due to the characteristics of the KHPS, there is a limit in identifying the context of the relationship between the occurrence of medical needs and unmet medical care; future research will require further analysis on this. Second, because major variables were derived from Andersen’s behavioral model, other external factors could not be included in the study. It is necessary to analyze changes in factors affecting the occurrence of unmet medical care for the patients with BPE by including family factors, external factors, etc., in addition to influencing factors according to Andersen’s behavioral model. Third, the occurrence of unmet medical care by type of medical institution was not examined. The quality of medical services will be different depending on the type of medical institution; therefore, the satisfaction of patients’ medical needs will be different. Future studies should analyze the occurrence of unmet medical care according to clinic- and hospital-level medical institutions to improve the quality of medical services.

## 5. Conclusions

This study attempted to analyze the factors influencing the unmet medical care of patients with BPE by using national data and suggesting a method to meet the medical needs of patients with BPE. In order to satisfy their medical needs, it is necessary to induce patients to seek early treatment by providing accurate information about the disease. In particular, medical needs must be met by providing ex ante treatment for households receiving medical benefits. In cases where patients often ignore their own health problems, such as BPE, policies should be enacted to raise awareness. Efforts such as health lectures on the prevention of BPE and the importance of early diagnosis are needed. Importantly, as chronic diseases, such as BPE, require continuous management, unmet medical care may be resolved by utilizing primary medical care. Through a community care project centered on primary medical care, there will be continuous management of diseases, and unmet medical care may be reduced by reinforcing medical accessibility for households receiving medical benefits.

## Figures and Tables

**Table 1:** Characteristics of the patients (*n* = 3003).

Characteristic	Category (Mean)	*n*	%
Age	<65 (50.35)	2154	71.7
	≥65 (77.49)	849	28.3
Education	<High school	622	20.7
	≥High school	2381	79.3
Marital status	Yes	2556	85.1
	No	447	14.9
Income	<300	1885	62.8
	≥300	1118	37.2
Job type	Salaried employee	1855	61.8
	Self-employee	1148	38.2
Insurance type	National health insurance	2961	98.6
	Medical aid	42	1.4
Experienced lying in a sickbed	Yes	257	8.6
	No	2746	91.4
Activity limitation	Yes	105	3.5
	No	2898	96.5
Subjective health status	Good	2415	80.4
	Poor	588	19.6
Chronic disease	Yes	989	32.9
	No	2014	67.1
Unmet medical needs	Yes	824	27.4
	No	2179	72.6

**Table 2:** Relationship between factors influencing medical services and unmet medical needs.

Characteristic	Type	Unmet Medical Needs				*p-* Value
		No	%	Yes	%	
Age	<65	1549	71.9	605	28.1	0.111
	≥65	630	74.2	219	25.8	
Education	<High school	423	68.0	199	32.0	0.003 **
	≥High school	1756	73.8	625	26.2	
Marital status	Yes	1848	72.3	708	27.7	0.241
	No	333	74.0	116	26.0	
Income	<300	1343	71.2	542	28.8	0.020 **
	≥300	836	74.8	282	25.2	
Job type	Salaried employee	1366	73.6	489	26.4	0.051 *
	Self-employee	813	70.8	335	29.2	
Insurance type	National health insurance	2156	72.8	805	27.5	0.010 **
	Medical aid	23	54.8	19	45.2	
Experience of lying in a sickbed	Yes	203	79.0	54	21.0	0.008 **
	No	1976	72.0	770	28.0	
Activity limitation	Yes	78	74.3	27	25.7	0.391
	No	2101	72.5	797	27.5	
Subjective health status	Good	1749	72.4	666	27.6	0.386
	Poor	430	73.1	158	26.9	
Chronic disease	Yes	717	72.5	272	27.5	0.495
	No	1462	72.6	552	27.4	

* *p* < 0.1, ** *p* < 0.05.

**Table 3:** Factors affecting unmet medical needs.

Independent Variables		Unmet Medical Needs					
		Model 1		Model 2		Model 3	
		B	Exp (B)	B	Exp (B)	B	Exp (B)
Predisposing Factors	Age	−0.406 ***	0.666	−0.450 ***	0.638	−0.446 ***	0.640
	Education	−0.523 ***	0.593	−0.456 ***	0.634	−0.477 ***	0.621
	Marital status	0.122	1.129	0.132	1.141	0.135	1.144
Enabling Factors	Income			−0.132	0.877	−0.125	0.882
	Job type			−0.115	0.892	−0.120	0.887
	Insurance type			−0.743 **	0.476	−0.773 **	0.462
Need Factors	Experience of lying in a sickbed					−0.384 **	0.681
	Activity limitation					0.066	1.068
	Subjective health status					−0.109	0.897
	Chronic disease					0.048	1.049
F(*p*)		21.173 ***(0.000)		31.163 ^***^(0.000)		38.551 ***(0.000)	

** *p* < 0.05, *** *p* < 0.001.
